# Examination of viability and quality of ovarian tissue after cryopreservation using simple laboratory methods in ewe

**DOI:** 10.1186/1477-7827-9-78

**Published:** 2011-06-08

**Authors:** Ghaya Merdassi, Claire Mazoyer, Jean F Guerin, Ali Saad, Bruno Salle, Jacqueline Lornage

**Affiliations:** 1 Laboratoire de Biologie de la reproduction, Université Claude Bernard Lyon 1, 43 Boulevard du 11 Novembre, 69100 Villeurbanne, France; 2 Unité de Procréation Médicalement Assistée Hôpital Aziza Othmana. Place Du Gouvernement 1000 Tunis, Tunisie; 3 INSERM U846, H Kennedy, 18 avenue Doyen Lépine, 69500 Bron, France; 4 Service de Médecine de la Reproduction, Hopital Femme Mère Enfant, 59 Bd Pinel, 69500 Bron, France; 5 Laboratoire de cytogénétique et biologie de la reproduction, Hôpital Farhat Hached, Sousse, Tunisie

## Abstract

**Background:**

The objective of the present study is to assess viability tests and to evaluate follicle ovarian tissue quality after freezing-thawing procedures.

**Methods:**

Ewe's ovaries were harvested at the slaughterhouse, after dissection each ovarian specimen was divided into two groups: fresh tissue (control group) and frozen tissue.

In the first part of the study, the follicles viability was assessed by trypan blue staining, calcein AM/ethidium homodimer-1 staining (LIVE/DEAD viability/cytotoxicity kit, Molecular Probes) and morphology in the two groups. In the second part of the study the quality of the whole ovarian tissue was evaluated by the quantification of the release of lactate dehydrogenase measurement (Cytotoxicity Detection kit ROCHE), DNA fragmentation by terminal deoxynucleotidyl transferase-mediated dUTP-biotin nick end labelling (TUNEL) in primordial and primary follicles (ApopDETEK Kit system Enzo) and morphology in the two groups. 100 Follicles (primordial and primary) were counted on both fresh and frozen hemiovary to assess this various tests.

**Results:**

Ovarian follicle viability assessment was similar using trypan blue or calcein/ethidium staining. Follicles showed a decreased viability after freezing-thawing.

After cryopreservation, a significant correlation between the percentage of normal follicles and viability rate was found using trypan blue (r = 0.82, p < 0.05) or calcein AM/ethidium homodimer-1 staining (r = 0.76, p < 0.05). Increased cytotoxicity showed by enhancement of LDH release was found after cryopreservation (21.60 +/- 1.1% vs 52.2 +/- 7.7%). A significant negative correlation between the percentage of morphologically normal follicles and cytotoxicity was observed. No significant difference in DNA fragmentation rate between frozen and control groups was found (26 ± 8.2% vs 38 ± 4.5%).

**Conclusion:**

We suggest the use of trypan blue staining for the histological assessment of viability, the use of LDH assay for the cytotoxicity assessement and finally the use of DNA fragmentation assessment to valid different freezing-thawing protocols.

## Background

Preserving the fertility of women or girls undergoing intense radio-and/or chemo-therapy remains a big challenge for the practitioners. Although, these therapeutic options are the only hope for those patients to heal, they remain very aggressive for germ cells causing destruction of ovarian tissue and leading to definitive secondary sterility. Different techniques and protocols of cryopreservation have emerged. Some teams, tried oocytes harvesting for cryopreservation. Others teams, opted for the ovarian tissues cryopreservation. This technique holds out the hope of restoring gonad function in iatrogenically sterilized cancer patients after autograft [1]. Animal studies of ovarian cryopreservation have enabled pregnancy after ovarian freezing-thawing and autograft in rat [2], mouse [3,4] and sheep [5,6] more over, some studies reported recovery of cyclic secretion of ovarian steroids [7]. Primordial follicles are less sensitive to the freezing procedure than growing follicles. They have smaller cells, with a low metabolic activity which may facilitate post-transplant recovery of ovarian function. The most common strategy is to conserve the primordial follicle population by freezing ovarian cortex fragments harvested at any point in the cycle by straightforward laparoscopy. In humans, live births have been obtained by frozen ovarian cortex fragment autograft [8]. Restored ovarian cycle has also been reported following transplantation of a frozen ovarian cortex [9]. Despite the progress in ovarian tissues cryopreservation, live births remain rare. It is important to improve freezing protocols to cryopreserve the existing ovarian reserve in perfect structural and functional condition. Improvements in surgical technique will also be necessary to reduce the delay in the recovery of steroidogenesis due to hypoxia preceding revascularization [10].

To assess the structural integrity and follicles viability of cryopreserved ovarian tissue, there are histological evaluation (by optical or electron microscopy) and vital staining [11-14]. To assess the functional integrity of cryopreserved ovarian tissue there are biochemical markers of cytotoxicity (such as lactate dehydrogenase (LDH) release measurement) and oocyte or granulosa cell DNA fragmentation assay [15-18].

The purpose of the present study was to evaluate the quality of ovarian tissue before and after freezing using a combination of upper mentioned techniques. Ewes were chosen as model due to their anatomic and physiological similarities of their ovaries comparing to those of human. Folliculogenesis is also relatively similar in both species.

We first compared viability in follicles isolated by two methods: the calcein AM/ethidium homodimer-1 which is a fluorescent marker. It is considered as the gold standard but 100 times more expensive than the trypan blue [13]. Then, we studied tissue integrity, using a biochemical assay of LDH and by follicle DNA fragmentation using the TUNEL assay. Results were assessed against the gold-standard of histological study of ovarian reserve (primordial, intermediate and primary follicles).

## Methods

### Collection of ovarian tissue

Ewes ovaries were harvested at the slaughterhouse immersed in a solution of X VIVO medium (Bio Whittaker, Walkersville, MD) at 4°C and transported to the laboratory within 45 minutes to limit warm ischemia. Each ovarian specimen was dissected sagittally and the medulla was removed. Approximately 1 mm-thick cortex was obtained and divided into two groups: fresh tissue (control group) and frozen tissue.

### Freezing and thawing of ovarian tissue

The cryopreservation procedure was based on a method described previously [7]. Hemi-ovaries of different ewes were frozen at the same time for each experiment. Briefly, ovarian cortexes were placed in straws (CryoBioSystem, Paris, France) containing 800 μl BM1 (Ellios Bio Media, Paris, France) freezing medium supplemented with 10% fetal calf serum and 2M dimethyl sulfoxide (DMSO) (Sigma). After 10 minutes' room-temperature incubation, the straws were placed in the automated freezer (Minicool 40 PC, Air Liquide Santé, France), cooled first at 2°C/min to -11°C and then from -40°C down to -140°C at a cooling rate of 25°C/min. Ice nucleation was induced at -11°C by injecting -15°C frigories. Freezing curves were saved to a computer. The straws were transferred to liquid nitrogen and kept for 24 hrs.

After rapid thawing at 37°C, the ovarian tissue was washed in BM1 twice for five minutes with mild agitation at room temperature to eliminate the DMSO cryoprotectant.

### Follicle viability

10 ovaries were harvested and divided to provide one frozen hemi-ovary and one fresh control. A fragment of control tissue was fixed in Bouin's liquid for histology, and the rest was dissociated immediately to determine the follicle viability rate, using trypan blue (Sigma-Aldrich, St. Louis, MO) and calcein AM/ethidium homodimer 1 (Molecular Probes) staining. After thawing, the frozen ovarian fragment underwent the same treatment as their fresh controls.

#### 1. Follicle isolation

The ovarian fragments were sliced in Leibovitz L-15 medium plus 1 mg/ml (200 U/ml) type I collagenase (Sigma-Aldrich, St. Louis, MO) and incubated at 37°C for 2 hrs and pipetted every half-hour. Collagenase was then inhibited by 50% fetal calf serum, and the suspension was passed through a 60 μm nylon filter and centrifuged at 2,500 rpm for 5 min. The pellet was diluted in 50 μl Leibovitz L-15 medium and kept in the incubator at 37°C for viability testing.

#### 2. Trypan Blue coloration

Follicles were stained using 0.4% trypan blue and examined under an inverted microscope (x 400 magnification). The follicles were identified as degenerated (blue-stained) and surviving (unstained). 100 small follicles were counted per high power field.

#### 3. Calcein AM/Ethidium homodimer-1 coloration

Ethidium homodimer-1 inserts in DNA, staining the cell nucleus red in case of membranolysis. Calcein AM is non-fluorescent but, after passing through a cell membrane, is converted into intensely fluorescent calcein by cellular esterase in the lysosomes, producing fluorescent green staining. The LIVE/DEAD Viability/Cytotoxicity Kit (Molecular Probes) used 5 μM ethidium homodimer-1 and 2 μM calcein. The pellet was diluted in 50 *l of the working solution and incubated at 37°C for 45 min in the incubator. Fluorescence was observed simultaneously using 485 ± 10 nm and 530 ± 12.5 nm optic filters for calcein AM and ethidium homodimer 1, respectively. Follicles were classified according to oocyte staining:

- Degenerated follicles: degenerated oocytes and degenerated or surviving follicular cells (red); surviving follicles: surviving oocytes and follicular cells (green); 100 small follicles were counted per high power field

### Tissue integrity

Each ovary was divided in two parts. From the control part, one fragment of tissue was fixed in Bouin's liquid for histology and another in 2% paraformaldehyde (PFA) for immunostaining. Two other fragments were used for LDH assay. After thawing, followed by 15 min incubation in BM1 before any manipulation, the frozen fragment underwent the same treatment as the fresh controls.

#### 1. LDH assay

A cytotoxicity detection kit (Roche, Penzberg, Germany) was used for the cytoplasmic enzyme activities released into the ovarian tissue culture supernatant in fresh tissue and after thawing. We first began by standardizing LDH assay on ovarian cortex fragments by determining the optimal culture medium, fragment size and assay times. We chose to use ovarian tissue fragments incubated in a medium based on DMEM supplemented according to Hovatta's protocol [19], which allows tissue survival without interference in the LDH assay. Two 7 mm² fragments from each ovary were placed in X VIVO medium. One served as control after homogenization in liquid nitrogen to release its cytosolic content.

The fragments were incubated with their respective controls for 2 hrs in 4-well culture plates (Nunc, Denmark) in DMEM/Ham's F-12 medium (Sigma-Aldrich, St. Louis, MO) supplemented with ITS+1 (10 mg/L insulin, 5.5 mg/L transferrin, 5 μg/L selenium, 0.5 mg/L BSA and 4.7 μg/L linoleic acid) (Sigma), 1.25 mg/ml BSA (Sigma), 50 μg/ml streptomycin (Sigma), and 75 μg/ml penicillin-G (Sigma). The plates were brought to equilibrium with 1 ml of culture medium and incubated at 37°C. The release of the enzyme lactate dehydrogenase from lysed cells was measured and the percentage of cell-mediated lysis was expressed as according to the following formula

Sample OD: culture media after the incubation of ovarian fragments

White OD: reagent blank

Control OD: culture medium after incubation of total damaged ovarian fragments

#### 2. TUNEL Technique

Ovarian fragments were fixed in 2% PFA, cut into 6- μm slices and placed on silanized slides. DNA fragmentation was studied by the TUNEL technique, using an ApopDETEK kit (Enzo Diagnostics, Farmingdale, NY). The slides were deparrrafinized in a 60°C bain-marie, in two xylene baths and rehydrated in decreasing alcohol baths.

**Figure 1:**
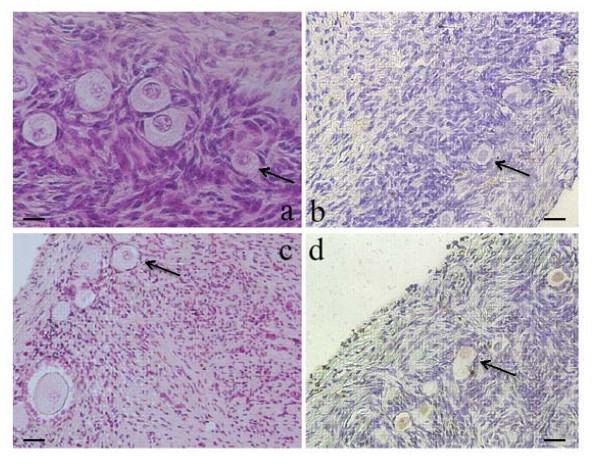
**A representative picture of ovarian cortex**. (A): A section of non-frozen control tissue containing primordial follicles, (B): A section of non-stained follicles in fresh ovarian cortex. (C): A section of primordial, intermediary follicles after ovarian tissue cryopreservation. (D): a section of follicles considered as TUNEL-positive with stained nuclei (brown) after cryopreservation. × 200 magnification. Arrows, primordial follicles 20 μm.

The slides were incubated with proteinase K at 37°C for 15 minutes for the ovary sections and for 10 minutes for the positive controls, before rinsing in PBS. Endogenous peroxidases were deactivated by 3% hydrogen peroxide (H_2_O_2_). Transferase Terminal staining was performed after incubating the slides in equilibrium buffer, then with terminal deoxynucleotidyl transferase (TdT) diluted in label reagent (Bio-16-dUTP) at 37°C for 60 minutes. The negative control slides were incubated in PBS. Detection was performed using streptavidin/ biotin/peroxidase at 37°C for 60 minutes followed by incubation in diaminobenzidine (DAB) for 18 minutes. Counter-staining with Mayer's hematoxylin (dilution 1/5) revealed non-stained follicles. DNA fragmentation was based on oocytes and follicular cells. Follicles with stained nuclei (brown) were considered TUNEL-positive (Figure 1).

### Histology

A histological study was carried out for each protocol. Ovary fragments were fixed in Bouin's liquid for at least 2 hrs and cut into 4 μm slices every 60 μm by microtome (Lietz, Germany). 100 primordial, intermediate and primary follicles with oocytes having visible nuclei were studied per group. All sections were examined microscopically at × 400 magnification to determine the percentage of "normal" follicles: those with normal follicular cell layer, cytoplasm and nucleus. Follicular anomalies were classified as nuclear (oocytes with pycnotic nuclei), cytoplasmic (intracytoplasmic vacuoles and/or deformed oocyte contour with basal membrane detachment) or mixed (both nuclear and cytoplasmic).

### Statistical analyses

Statistical analysis used SPSS.12.3 software. As our series were matched, a Wilcoxon test was applied. As dispersion showed a linear distribution, Pearson's coefficients were calculated to maximize power. The two tissue-study series were then compared by non-parametric Mann-Whitney U test. The significance threshold was set at p < 0.05.

## Results

### Follicle viability

There was no significant difference in estimated follicle viability with trypan blue and calcein AM/ethidium homodimer-1 staining, respectively, 76 ± 9.7% and 79 ± 9.7%; (correlation coefficient = 0.96 (p < 0.001)) in the control group and respectively, 61 ± 13% and 65 ± 12.5%; (correlation coefficient = 0.98 (p < 0.001)) in the frozen tissue.

The percentage of morphologically normal follicles correlated significantly with viability, for both trypan blue (r = 0.82, p < 0.05) and calcein AM/ethidium homodimer-1 (r = 0.76, p < 0.05).

### Tissue integrity

The percentage of cytotoxicity as indicated by LDH release in the whole ovarian tissue increase significantly after freezing 21.60 ± 1.1% vs 52.2 ± 7.7% ( p < 0.05). There was no correlation between normal follicles, morphology and percentage of cytotoxicity in fresh tissue. There was a significant negative correlation between normal follicles morphology and cytotoxicity percentage after freezing (r = -0.89, p < 0.05).

TUNEL DNA fragmentation study found no labeling was observed on the negative controls. There was no significant difference between control and freezing groups respectively 26 ± 8.2% and 38 ± 4.5%; (Table 1). The semi-quantitative follicular cell DNA fragmentation study showed no change with freezing (Figure 1).

**Table 1:** Percentage of follicles with positive DNA fragmentation on fresh and frozen-thawed ovarian tissue

	Fresh ovaries	Frozen Ovaries
**% of positive DNA fragmentation in oocytes**	**26 ± 8,2**	**38,5 ± 4,5**
**Positive DNA fragmentation in follicular cells**	**+**	**++**

Note: Values are means ± SD

**+: **5-25% Follicular cells TUNEL

+ **++: **< 25% Follicular cells TUNEL+

There was a significant negative correlation between the percentage of follicle DNA fragmentation and normal follicle morphology in fresh tissue (r = -0.96, p < 0.05). After freezing, the correlation disappeared.

## Discussion

In many malignant diseases, chemotherapy and/or radiotherapy treatment are the only hope for young girl or woman to survive their disease. At the same time, those therapeutic options induce definitive sterility by destroying ovarian reserve and/or follicles in growth-phase. To preserve the fertility of such patients, their ovarian cortex may be harvested and cryporeserved pending future use [1,19,20]. Indeed the ovarian cryopreservation started to be proposed as a therapeutic aid to preserve fertility; it still raises a number of questions. One major issue is to assess the quality of the cryopreserved ovarian tissue and follicles before an eventual use [21-23].

The present study developed and compared tests of follicular survival and quality, using a fragment of tissue to assess the ovarian cortex before and after freezing.

The first phase of our investigation concerned isolated follicle viability by comparing two staining techniques (trypan blue and calcein AM/ethidium homodimer-1) against a morphological gold-standard [24]. Trypan blue acts as an exclusion test, not penetrating live cells with intact membrane structures [14]. Ethidium homodimer-1 inserts in the DNA, staining the nucleus red when the membrane is broken. Non-fluorescent calcein AM is converted into intensely fluorescent calcein by cellular esterase in the lysosomes, staining live follicles green when the calcein AM passes through the cell membrane [15].

The two techniques showed a comparable viability rates before and after freezing. The histological assessment confirmed these findings. Our results showed that trypan blue test to provide the same information as calcein AM/ethidium homodimer-1, at one hundredth part of the cost. Our findings were in agreement with Fauque *et al*., who demonstrated the interest of trypan blue for the assessment of follicular viability [25]. Viability and morphology provide a first assessment of follicular status, using small fragments of ovarian tissue [26-28]. The second phase of our investigation is to check the resistance of ovarian tissue to freezing and to quantify viability of stromal and folliclar cells. LDH assays have been previously reported as an indicator for tissue integrity in cell culture; however there are few reports for its use with ovarian cortex fragments. Some authors assayed LDH in ovarian tissue culture medium, or after complete homogenization of ovarian fragments [16,17]. We were able to assay LDH on small fragments of 5-8 mm^2 ^with a thickness of 1 mm [19].

Various times assay were initially tested; variations in optical density were observed as of 2 hrs incubation. According to Cirelli *et al*., 80% of LDH release occurs during the first 3 hours of incubation [29]. This supports our approach, assessing each fragment for 2 hours in the medium to quantify cytotoxicity after freezing

The pre-incubation equilibration phase of tissues in BM1 medium before assaying LDH seems to be important, as the mechanical manipulation of the tissue to remove the medulla tends to increase LDH release. According to Terry *et al*., an equilibration phase is generally required to stabilize release of biochemical markers [30]. LDH assay results were expressed as percentage cytotoxicity. Damage to each frozen sample was quantified with respect to the corresponding fresh control [16].

LDH released into the incubation medium marks cytoplasmic membrane rupture, and is thus related to cell death. There is no correlation between normal follicle count and the percentage of cytotoxicity in fresh tissue due to the stabilization period of fresh tissue before LDH assays The increase of cytotoxicity percentage was negatively correlated with normal follicle count during freezing, it's clear that LDH assay is not only a marker of follicles integrity but also of stromal and follicular cells due to membrane rupture in ovarian tissue cells. Previous study showed that the stroma shows cryo damages signs after slow programmed freezing which increase cytotoxicity [31]. LDH assay is a low-cost technique, using small ovarian tissue fragments and providing rapid assessment of the tissue. That's why it is of great interest to optimize this method to illustrate the changes in the ovarian stromal tissue caused by different cryopreservation protocols and a complement to viability and morphological assessment in cryopreserved tissue.

DNA fragmentation is the final phase of apoptosis. It is therefore preferable to use fragmentation, as distinct from apoptosis [32]. There are various techniques for revealing fragmentation: COMET, TUNEL and Acridine Orange staining. We chose the TUNEL technique because it could be implemented on slices, which is not the case with the COMET technique, and was easier to interpret than acridine orange staining. Semi-quantitative analysis assessed fragmentation in the follicular cells surrounding each oocyte in the fresh and frozen tissue samples.

Labeled follicles were present in fresh tissue and in frozen thawed tissue (26 ± 8.2% and 38 ± 4.5%). In fresh tissue labeled follicles shows the presence of atretic small follicles.

Lee *et al*., reported 32.5% of apoptosis for primordial and primary follicles in mouse ovary [23]. There was no significant increase in follicle DNA fragmentation after freezing, with the present protocol, immature follicles can thus be cryopreserved without irreversible damage to their DNA. A significant negative correlation between follicle DNA fragmentation and the percentage of normal follicles in fresh tissue was observed but no correlation was found after freezing. This observation supports the hypothesis that a proportion of cells are lost following cryopreservation due to necrosis [21,33].

One of the main aims of our study was to obtain a clearer picture of ovarian tissue cryopreservation effects using rapid tests such as Tunel technique. Authors have associated follicle DNA fragmentation study to Bax and caspase-3 apoptosis protein assay [25,34,35]. We preferred to correlate our fragmentation test with a valid method such as histo-morphological assessment. These results suggest that cryopreservation of human ovarian tissue is feasible. However, it's necessary to look for follicles density which can gives much more information about structural damage and cells loss.

## Conclusion

A slow-freezing protocol has proven to be an effective alternative method in preservation of fertility. However, a simple and convenient technique is required for assessment of cell survival in ovarian cryopreserved tissue. In fact, since the major aim of cropreservation technique is to restore fertility after iatrogenic sterilizations, it is easy to conclude that it is crucial to validate the freezing protocol in order to evaluate the success of xeno-or auto-graft transplantation of frozen-thawed ovarian fragments, before involving the patient in an onerous procedure [36-38].

The present study pointed some easy and inexpensive tests which can be used in routine practice for the assessment of follicular function before and after freezing. To reach this aim we suggest:

1- Histological study with viability assessment by trypan blue staining

2- LDH assay to quantify whole tissue death to evaluate the whole ovarian tissue

3-Tunel technique to evaluate DNA fragmentation for the validation of different freezing protocol.

## Competing interests

The authors declare that they have no competing interests.

## Authors' contributions

GM carried out the viability tests and the evaluation of follicle ovarian tissue quality after freezing-thawing and drafted the manuscript. CM carried out the different tests and the histology studies. JFG participated in the coordination of the study. AS participated in the design of the study. BS helped to draft the manuscript. JL conceived of the study and participated in the design and the draft

All authors read and approved the final manuscript.
